# Phytochemicals Modulate Biosynthesis and Function of Serotonin, Dopamine, and Norepinephrine for Treatment of Monoamine Neurotransmission-Related Psychiatric Diseases

**DOI:** 10.3390/ijms26072916

**Published:** 2025-03-23

**Authors:** Makoto Naoi, Yuqiu Wu, Wakako Maruyama, Masayo Shamoto-Nagai

**Affiliations:** Department of Health and Nutritional Sciences, Faculty of Health Sciences, Aichi Gakuin University, 12 Araike, Iwasaki-cho, Nisshin 320-195, Aichi, Japan; wuaki@dpc.agu.ac.jp (Y.W.); maruyama@dpc.agu.ac.jp (W.M.); nagaim@dpc.agu.ac.jp (M.S.-N.)

**Keywords:** serotonin, catecholamines, neurotransmission, phytochemicals, tryptophan hydroxylase, tyrosine hydroxylase, monoamine oxidase, neuropsychiatric diseases

## Abstract

Serotonin (5-HT), dopamine (DA), and norepinephrine (NE) are key monoamine neurotransmitters regulating behaviors, mood, and cognition. 5-HT affects early brain development, and its dysfunction induces brain vulnerability to stress, raising the risk of depression, anxiety, and autism in adulthood. These neurotransmitters are synthesized from tryptophan and tyrosine via hydroxylation and decarboxylation, and are metabolized by monoamine oxidase (MAO). This review aims to summarize the current findings on the role of dietary phytochemicals in modulating monoamine neurotransmitter biosynthesis, metabolism, and function, with an emphasis on their potential therapeutic applications in neuropsychiatric disorders. Phytochemicals exert antioxidant, neurotrophic, and neurohormonal activities, regulate gene expression, and induce epigenetic modifications. Phytoestrogens activate the estrogen receptors or estrogen-responsive elements of the promoter of target genes, enhance transcription of tryptophan hydroxylase and tyrosine hydroxylase, while inhibiting that of MAO. These compounds also influence the interaction between genetic and environmental factors, potentially reversing dysregulated neurotransmission and the brain architecture associated with neuropsychiatric conditions. Despite promising preclinical findings, clinical applications of phytochemicals remain challenging. Advances in nanotechnology and targeted delivery systems offer potential solutions to enhance clinical efficacy. This review discusses mechanisms, challenges, and strategies, underscoring the need for further research to advance phytochemical-based interventions for neuropsychiatric diseases.

## 1. Introduction

Aromatic amino acids in the brain serve as precursors for the monoamine neurotransmitters, with tryptophan giving rise to serotonin (5-hydroxytryptamine, 5-HT) and tyrosine serving as the substrate for catecholamines, including dopamine (DA), norepinephrine (NE), and epinephrine (E). Monoamine neurotransmitters include indoleamines and catecholamines, and trace amines. They not only transmit neuronal signals, but also have pleiotropic activity, regulate neuronal function, structure, and are implicated in the pathogenesis of neuropsychiatric disorders. As the predominant neurotransmitter among indoleamines, 5-HT regulates brain development via neuronal differentiation, proliferation, synaptogenesis, dendrite organization, and neurogenesis, and it affects mood, anxiety, stress, sexual behaviors, and cognition [1,2]. 5-HT dysfunction during early brain development impairs the synaptic network and impacts the outcome of schizophrenic psychoses, the autism spectrum, and attention-deficit/hyperactivity disorder (ADHD) [3]. The catecholamines DA and NE play key roles in motor function, spatial memory, motivation, arousal, reward, and pleasure. Another catecholamine, E, has a more limited role in central nervous system (CNS) function but is primarily involved in peripheral sympathetic responses. Malfunctions of DA signaling are implicated in the pathogenesis of Parkinson’s disease (PD), Huntington’s disease (HD), ADHD, and schizophrenia [4]. Deficits in monoamines have been targeted for the treatment of depression and PD and beneficial results have been presented.

Monoamine neurotransmission is a complex process, including monoamine biosynthesis, storage, release, interaction with its specific post- and pre-synaptic receptors, uptake, and catabolism [5]. In the brain, indolamines and catecholamines are synthesized and metabolized by similar enzymatic mechanisms. 5-HT and DA are synthesized from *L*-tryptophan and *L*-tyrosine, in serotonergic or catecholaminergic neurons, respectively, by two-step reactions: in serotonergic neurons, tryptophan hydroxylase (TPH) catalyzes the hydroxylation of *L*-tryptophan, followed by decarboxylation by aromatic amino acid decarboxylase (AADC) to produce 5-HT; in catecholaminergic neurons, tyrosine hydroxylase (TH) catalyzes the hydroxylation of *L*-tyrosine to *L*-DOPA, which is subsequently decarboxylated by AADC to form DA [6,7]. NE is then synthesized from DA by dopamine-β-hydroxylase (DBH), and E is synthesized from NE by phenylethanolamine-*N*-methyltransferase (PNMT). Monoamines are metabolized mainly by monoamine oxidase (MAO) and catechol *O*-methyltransferase (COMT).

Multiple genetic and environmental factors modulate the expression and activity of monoamine-related enzymes and strictly regulate monoamine levels in the brain. TPH and TH activity are regulated by phosphorylation by protein kinases, and feedback inhibition by the monoamines and allosteric regulation [8]. Stress influences TPH and TH expression at multiple stages [9]. Expression of neuron-specific TPH2 is mainly regulated by stress, cortisol, and 5′- and 3′-regulatory polymorphisms of *TPH2* [10]. TH and TPH expression and activity are regulated by estrogen and glucocorticoids [11]. Diet, vitamins, and nutraceuticals modulate monoamine biosynthesis and the metabolism of 5-HT in the brain. The 5-HT biosynthesis rate mainly depends on the brain’s *L*-tryptophan levels [7,12]. AADC requires pyridoxal phosphate, DBH ascorbic acid, and PNMT *S*-adenosylmethionine (SAM) as a methyl donor for enzymatic activity.

Diet affects age-related neurodegeneration and cognitive decline, and ingredients of plant food have been proposed as neuroprotective molecules [13]. Phytochemicals are secondary plant metabolites and polyphenols are a major member, including flavonoids and several classes of non-flavonoids such as phenolic acids, stilbenes, and lignans [14]. These compounds exhibit various biological effects, such as antioxidant activity, regulation of cellular signaling and gene induction, neuroprotection, immune-regulation, and modulation of gut microbiota. Neuroprotective polyphenols modulate neurotransmission systems and ameliorate neurochemical and behavioral changes associated with neuropsychiatric disorders [15,16,17]. Some bioactive polyphenols, known as phytoestrogens, have biological activity similar to 17*β*-estradiol (E2). They bind to the estrogen receptors (ERs) and exert pro- and anti-estrogenic effects [18]. They modulate monoamine transmitter systems via affecting the expression and activity of related enzymes, TPH, TH, AADC, and MAO [19].

This paper presents the therapeutic potential of phytochemicals against neurobehavioral disorders caused by deregulated homeostasis of monoamine neurotransmission. The genetic and environmental factors regulate monoamine biosynthesis, metabolism, and function, and their deregulation in the neuropsychiatric disorders is discussed [1,20]. Multiple neuroprotective functions of polyphenols, such as antioxidant, anti-apoptosis functions, induction of pro-survival genes, and neurotrophic factor (NTF)-like activity are reviewed [21,22,23,24,25]. The epigenetic effects of phytochemicals on the interaction between genetic and environmental factors in the early phase of brain development is discussed. A deficit or excess of 5-HT levels in the developing brain is associated with depression, aggressive behaviors, and anxiety in adulthood. The strategy to apply phytochemicals for the prevention and therapy of neuropsychiatric diseases is discussed.

## 2. Biosynthesis, Metabolism, and Function of Monoamine Neurotransmitters and Related Enzymes in the Brain

**Figure 1:**
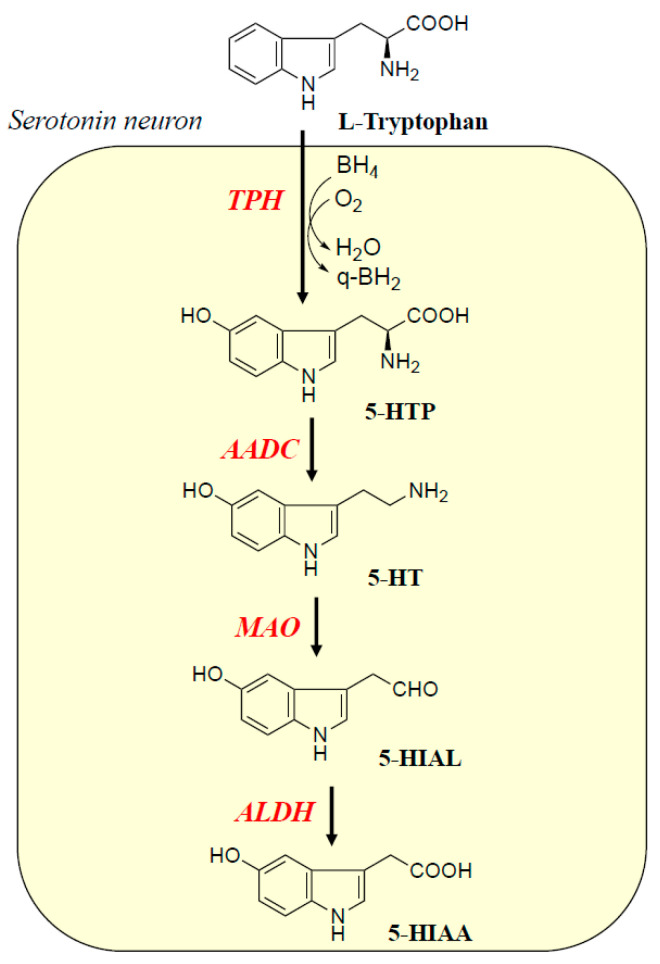
Biosynthesis and metabolism of 5-HT. TPH hydroxylates *L*-tryptophan to 5-hydroxytryptophan (5-HTP), which AADC decarboxylates to 5-HT. MAO-A oxidizes 5-HT to its aldehyde, which ALDH oxidizes to 5-hydroxyindolacetic acid (5-HIAA). TPH requires BH_4_ and O_2_ for hydroxylation (H_2_O) and quinonoid dihydrobiopterin (q-BH_2_).

5-HT is synthesized in only a small number of neurons localized in the midbrain dorsal and ventral raphe nuclei, but the 5-HT release site and synapses are distributed throughout the brain. TPH hydroxylates *L*-tryptophan into 5-hydroxytryptophan (5-HTP) using Fe^2+^, (6*R*)-*L*-tetrahydrobiopterin (BH_4_), and diatomic oxygen (O_2_) (Figure 1). 5-HTP is decarboxylated into 5-HT by AADC. 5-HT is metabolized by type A MAO (MAO-A) to 5-hydroxyindol-acetaldehyde (5-HIAL), which aldehyde dehydrogenase (ALDH) converts to 5-hydroxyindolacetic acid (5-HIAA). The 5-HT system is implicated in regulating human behaviors, state of mind, mood, social interaction, and consciousness, and is involved in the outcome of schizophrenic psychoses, the autism spectrum, and ADHD [3].

TH and TPH are members of aromatic amino acid hydroxylase (AAAH) family monooxygenases and perform the hydroxylation of the aromatic ring of an amino acid by use of diatomic oxygen, a catalytic non-heme ferrous iron, and BH_4_ as a coenzyme [26]. They have very similar active sites and are composed of a multi-domain structure: the *N*-terminal regulator domain (R) of 100–150 amino acid residues, the catalytic domain (C) of about 330 residues, and a coiled-coil domain at the *C*-terminal of about 20 amino acids. There are two TPH isoenzymes, TPH1 and TPH2, encoded in different genes localized on chromosomes 11 and 12, respectively, with sequence identity of 71% [27]. TPH2 is expressed predominantly in the brain, whereas TPH1 in peripheral tissues. TPH2 contains a larger regulatory domain than TPH1, and an additional 41 amino acids at the *N*-terminus. A serine at position 16 (Ser16) of TPH2 is phosphorylated by cAMP-dependent protein kinase A (PKA) and increases the activity, interacts with 14-3-3 proteins, and increases protein stability [28]. There are more than 300 single-nucleotide polymorphisms (SNPs) of *TPH* that have been identified in humans, six are coding non-synonymous SNPs (L36P, P206S, A328V, R441H, D479E), and three are coding synonymous SNPs (P312P, L327L. A355). Some of these polymorphisms are associated with major depressive disorder (MDD), suicide, and obsessive-compulsive disorder [29,30], but other studies presented lack these associations [31]. 5-HT synthesis in the brain is highly limited by serum *L*-tryptophan levels.

**Figure 2:**
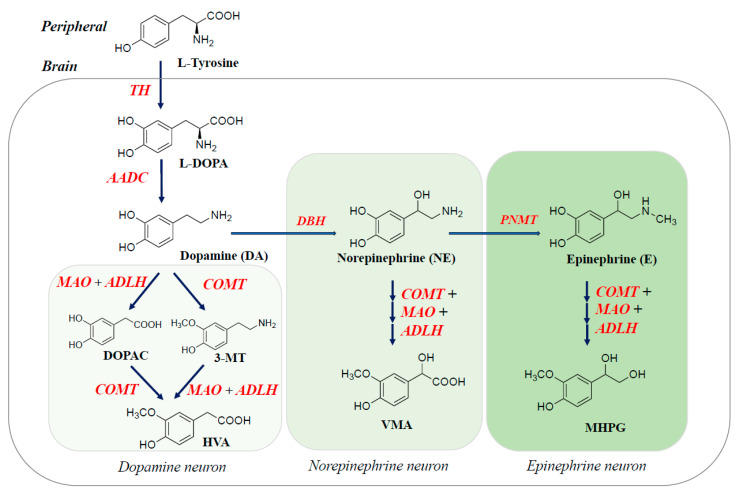
Biosynthesis pathway of catecholamine neurotransmitters in the brain. TH hydroxylates *L*-tyrosine to *L*-DOPA, which AADC decarboxylates to DA. DBH hydroxylates DA into NE, which is methylated to E by PNMT. DA are oxidized by MAO-A and MAO-B to the corresponding aldehydes and further oxidized by ALDH and *O*-methylates by COMT.

The DA neurons are mainly localized in the substantia nigra (SN) pars compacta, ventral tegmental area of the midbrain, and the hypothalamus. DA biosynthesis is regulated by the activity of TH and guanidine triphosphate cyclohydrolase I (GTPCH), the rate-limiting enzyme of BH_4_ biosynthesis. MAO-A and -B oxidize DA into 3,4-dihydroxyphenlacetaldehyde (DOPAL), which is oxidized by ALDH to 3,4-dihydroxyphenylacetic acid (DOPAC), or reduced by aldehyde reductase (ARL) to 3,4-htdroxyphenylglycol (DHPG). COMT converts DA into 3-methoxytyramine (3-MT) and NE, E and *L*-DOPA into normetanephrine (NMN), and metanephrine (MN) and 3-methoxydopa (3-MD), respectively (Figure 2).

The human TH (*hTH*) gene is localized on chromosome 11p15.5 and has 14 exons. The *hTH* is transcripted to alternative splicing at the 5′ end and produces four isoforms of *hTH* 1, 2, 3, 4 with different R domains [32]. TH activity is regulated by feedback inhibition via allosteric modulation by polyanions and posttranslational phosphorylation and dephosphorylation. DA, NE, and E are feedback inhibitors of TH via binding to the active site though the iron atom. Phosphorylation of Ser40 in the R domain by PKA increases the dissociation of catecholamines from the iron by 2–3 orders of magnitude, and decreases the Km value for BH_4_ by 2-fold [33]. The phosphatidylinositol-3 kinase (PI3K) and ERK1/2 pathways increased phosphorylation of Ser31 of TH, protein kinase C (PKC) Ser19, and PKA Ser40, respectively. Ser40 phosphorylation increased TH activity [34].

The TH protein is found to decrease markedly in the SN of Parkinsonian patients. TH mutations have been speculated to modify the susceptibility of the sporadic form of PD [35]. Three SNPs (rs2072056, rs6356, rs10743152) were reported within the *TH* gene, but the association with PD risk was not fully proven [32]. *hTH* deficiency is an autosomal recessive disorder due to mutations in the *TH* gene, and fewer than 40 patients were reported worldwide. According to neurological features, two phenotypes of TH deficiency were reported. Type A presents progressive hypokinetic-rigid syndrome with dystonia of infantile onset, whereas type B presents a complex encephalopathy of neonatal onset [36]. TH expression is induced by acetylcholine through nicotinic cholinergic receptors, and by vasoactive intestinal polypeptide (VIP) and pituitary adenylate cyclase-activating peptide (PACAP) in PC12 cells and sympathetic ganglia. The *TH* gene promoter contains cAMP responsive elements (CREs) and activates the protein-1 (AP-1) site [37], and *TH* transcription is regulated by PKA signal transduction [38].

The AADC catalyzes the decarboxylation of *L*-DOPA, 5-hydrotryptophan, and, much less efficiently, aromatic amino acids (tyrosine, tryptophan, phenylalanine) and histidine [39]. The *AADC* gene is localized in the catecholaminergic and serotonergic neurons of the central and peripheral nervous system and in chromaffin cells of the adrenal medulla. The human AADC is localized at 7p12.2-p.12.1 and has 15 exons and 14 introns [40]. Missense variants, frameshift variants, and alterations of splicing sites cause AADC deficiency. AADC deficiency is a rare congenital autosomal disorder, decreases 5-HT and DA biosynthesis, and causes neurodevelopment delay, hypotonia, oculogyric crises, and movement disorders [41].

MAO is classified into type A and B (MAO-A, MAO-B), according to substrate specificity and inhibitor sensitivity. MAO-A has a higher affinity to 5-HT, NE, and E, and MAO-B to phenylethylamine (PEA), benzylamine (BA), and octopamine. DA, tyramine, and tryptamine are common substrates for both MAO-A and -B. MAO-A is localized in catecholaminergic neurons and MAO-B in serotonergic and histaminergic neurons. Deregulated MAO-A activity is implicated in the pathogenesis of depression and antisocial behaviors, suggesting the possible application of a MAO-A inhibitor as a tool for treatment [42]. MAO-B increases with age and promotes neurotoxic reactive oxygen species (ROS), which is one of the pathogenic factors in PD and Alzheimer’s disease (AD). MAO-A regulates the 5-HT levels in an embryonic brain and modulates the development of brain architecture [43].

Human MAO-A and -B are coded by distinct genes localized on the X-chromosome (Xp11.23) and the *MAO-A* gene contains the *MAO-A* gene-linked polymorphic region (*MAO-A-LPR*) positioned about 1.2 kb upstream of the *MAO-A* transcription initiation site. The MAO-A variable number tandem repeat (*MAO-A-VNTR*) polymorphism codes 3, 3.5, 4, or 5 copies of the 30 bp repetitive sequence and affects the transcriptional activity of the *MAO-A* gene promoter [44]. Alleles with 3.5 or 4 copies of the repeated sequence (*MAO-A-H*) are transcribed 2–10 times more efficiently than 2, 3, or 5 copies (*MAO-A-L*). The *MAO-A* polymorphism drives variations in MAO-A activity and influences impulsivity and aggression. The *MAO-A-L* enhances the risk for developing an aggressive, antisocial personality. Low *MAO-A* expression causes 5-HT excess and impairs critical neural circuitry for social evaluation and emotion regulation, resulting in amplifying the effects of adverse early life experiments [45]. *MAO-A-H* carriers are at a greater risk of depression [46], but the results are still conflicting. A MAO-A deficit in Brunner syndrome caused by a nonsense mutation of the *MAO-A* gene (rs72554632) and Norrie disease causes behavioral abnormality, such as episodic impulsive aggression and borderline mental retardation [47,48].

COMT catalyzes the methylation of catechol substances, including catecholamines, catechol estrogens, and polyphenols, and is expressed throughout the brain. COMT exists in two forms: a membrane-bound form in the brain and a soluble form in the peripheral tissues. A membrane-bound COMT has higher affinity to DA in the human prefrontal neurons [49]. COMT plays a role in cognition, behavior, emotion, pain processing, addictive behavior, and neurodegeneration, and is implicated in neuropsychiatric disorders in a sex-different way [50,51]. The *COMT* gene is localized in chromosome 22q11, one of the principal loci linked to schizophrenia. Val158Met functional polymorphism plays a primary role in DA metabolism in the prefrontal cortex, and is associated with negative symptoms in patients with schizophrenia and bipolar disorder [52].

## 3. Neuroprotective Effects of Phytochemicals in Brain Health

**Figure 3:**
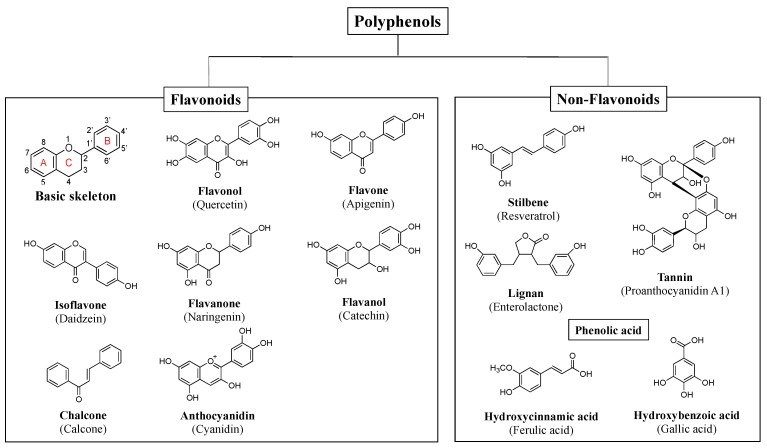
Classification and chemical structures of major phytochemicals discussed in the text.

Phytochemicals have multiple neuroprotective activities, such as antioxidant, anti-inflammatory, antiapoptotic activity, mitochondrial stabilization, activation of cellar signaling pathway, and induction of neuroprotective genes [22,53]. Some of them can cross the blood–brain barrier (BBB) and show protective function in neurodegenerative disorders [54]. The phytochemical superfamily consists of polyphenols (flavonoids, non-flavonoids), terpenoids/isoprenoids (saponins, lycopene, etc.) and nitrogen-containing alkaloids (caffeine, morphine, nicotine, etc.). Bioactive polyphenols are major phytochemicals composed of multi hydroxyl (-OH) aromatic phenols. Approximately 8000 polyphenols have been identified, and more than 4000 belong to flavonoids [55]. Flavonoids are subclassified into flavonols (quercetin, kaempferol, myricetin, etc.); flavones (luteolin, apigenin, etc.); isoflavones (daidzein, genistein, etc.); flavanones (naringenin, hesperetin, etc.), flavanols [catechin, epicatechin (EC), gallocatechin (GC), epigallocatechin (EGC), its gallate (EGCG), etc.], chalcone; and anthocyanidins (malvidin, cyanidin, etc.). Non-flavonoids include stilbene (resveratrol), lignan (enterolactone), tannin (Proanthocyanidin A1), and phenolic acids (ferulic, ellagic, tannic, gallic and caffeic acids, etc.) [14] (Figure 3).

**Figure 4:**
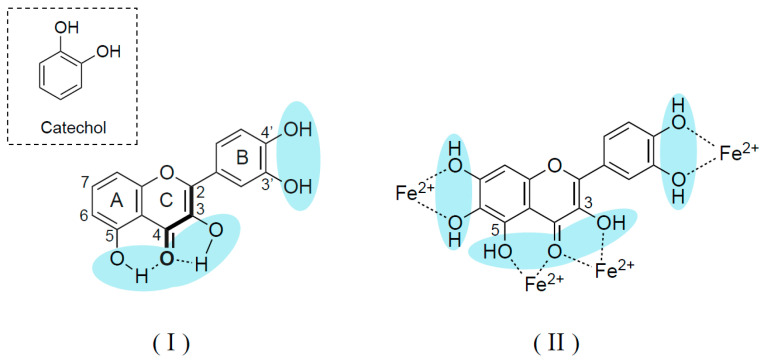
Structures required for antioxidant actions of flavonoids. (**I**); for radical scavenging (**II**); for metal chelating. The catechol group (3′, 4′-hydroxyl group) in the ring B, 2,3-double bond in conjugation with a 4-keto function in ring C and 3- and 5-hydroxyl group in rings C and A are essential structures for antioxidant function.

Oxidative stress and inflammation are common features of neurodegenerative disorders and aging. Polyphenols have broad antioxidant activity; scavenging free radicals, metal chelating, mitochondrial protection, and suppression of ROS-generating enzymes. The structure required for flavonoids and phenolic acids to scavenge radicals and chelate metals include the catechol group (3′, 4′-hydroxy groups in ring B, C2-C3 double bond in conjugation with a C4-keto function in ring C and presence of 3- and 5-hydroxyl groups in ring C and A) [56,57] (Figure 4). Flavonoids, curcumin, and caffeic acid alkyl esters regulate the gene expression of antioxidant enzymes [superoxide dismutase (SOD), catalase, glutathione reductase (GR), glutathione peroxidase (GPx)] by nuclear erythroid 2-related factor 2/antioxidant response element (Nrf2/ARE) signaling pathways [58,59]. Flavonols activate Nrf2, a transcription factor, and increase ARE-regulated genes.

Polyphenols activate PI3K, Akt/protein kinase B (Akt/PKB), tyrosine kinases, PKC and mitogen activated protein kinase (MAPK) signal pathways, and affect cellular function by modulating gene expression and phosphorylating targeted molecules [60,61]. Flavonoids selectively interact with MAPK signaling pathways to activate the expression of neuroprotective genes involved in NTF-induced differentiation, apoptosis, and various forms of cellular plasticity. Stress signals (DA, 4-hydroxy-2-nonenal, ROS, inflammatory kinases) activate c-Jun-*N*-terminal kinase (JNK) and p38, leading to apoptosis. Flavonols, epicatechin, and baicalein, suppress JNK and downstream c-jun and pro-caspase-3, and protect neurons.

Polyphenols exert NTF-like actions through direct binding to NTF-receptors, activating cellular signaling pathways, and inducing NTF expression [62,63]. 7,8-Dihydroxyflavone has a direct agonistic effect on the tropomyosin receptor kinase (Trk) receptor for the brain-derived neurotrophic factor (BDNF) and nerve growth factor (NGF). Flavonoids activate ERK and PI3K/Akt signaling pathways and increase NTF expression, whereas apigenin, ferulic acid, and resveratrol increase cAMP response element-binding protein (CREB) phosphorylation, and increase BDNF and glial cell line-derived neurotrophic factor (GDNF). High flavonoid intake induced serum BDNF levels, improved cognitive function in healthy subjects [64], and, in women with premenstrual syndrome [65] in randomized, double-blind, placebo-control trials, curcumin increased serum BDNF levels.

Polyphenols have been proposed to maintain mitochondrial homeostasis and ATP synthesis, modulate apoptosis systems in mitochondria, and protect neurons [23,66]. Resveratrol activated sirtuin-1 (SIRT1), decreased acetylation of peroxisome proliferator-activated receptor γ coactivator α (PGC-1α), increased genes for oxidative phosphorylation and mitochondrial biogenesis, and improved mitochondrial functions [67]. Mitochondria are directly involved in neuronal programmed cell death. Apoptosis progresses sequentially via the opening of the mitochondrial permeability transition pore (mPTP), resulting in the release of calcium and apoptogenic proteins [cytochrome c (Cytc), Smac/DIABLO, Omi] into cytosol, activation of caspases and subsequent chromatin condensation, and DNA fragmentation [68]. The mPTP is a complex apparatus mainly composed of the voltage-dependent anion channel (VDAC), the outer membrane transporter protein (TSPO), the Bcl-2 protein family at the outer mitochondrial membrane (OMM), the adenine nucleotide translocator (ANT) at the inner mitochondrial membrane (IMM), and cyclophilin D (CypD) at the matrix. By using the cellular model of apoptosis induced by a TSPO ligand, PK11195, the process of the mPTP opening was followed [69,70]. PK11195 opens a transitionally reversible pore at the IMM, declines mitochondrial membrane potential (ΔΨm), and allows the influx of water and molecules of less than 980 Da. Excess stimulus irreversibly forms the mPTP and releases molecules up to 1500 Da, such as Cytc. Phytochemicals such as ferulic acid, its derivatives, astaxanthin, and some flavonoids, prevent the pore formation of ANT with CypD at the IMM, thereby inhibiting mPTP formation and apoptosis [24]. Astaxanthin and resveratrol decreased the expression of CypD and ANT in heart mitochondria isolated from rats treated with isoproterenol [71] and after ischemia-reperfusion [72]. Resveratrol downregulated VDAC expression, dephosphorylated or deacetylated VDAC, prevented the mPTP opening induced by myocardial ischemia-reperfusion injury [73,74]. Curcumin binds to and stabilizes VDAC in the closed state, influencing mitochondrial function, apoptosis, and mPTP regulation [75]. However, quercetin has ambivalent redox activity and can inhibit or induce the mPTP [76]. (In the Supporting Information, Table S1 presents the chemical compounds and their function on affecting mPTP formation).

## 4. Sex- and Stress-Related Hormones Modulate Monoamine Neurotransmission in the Brain

The monoamine neurotransmitter system responds to extrinsic and intrinsic stimuli, and adapts its function and expression to maintain brain function [77]. Monoamine neurotransmission is regulated by sex-related estrogens and androgens (testosterone, dihydrotestosterone), and stress-induced corticoids, neurotransmitters, NTFs, ROS, and calcium [11]. Estrogens impact the cellular differentiation and neural network formation, including 5-HT and DA systems. E2 directly modulated promoter activity of *TPH2*, *5-HT transporter* (*SERT*, *SLC6A4*), *TH*, *DBH*, and *GTPCH* and increased expression [78,79]. Sex chromosomes (XX versus XY) and sex hormones (estrogen versus testosterone) are the factors for sex differences in neuropsychiatric disorders [80,81,82]. Stress activates the hypothalamic–pituitary–adrenal (HPA) axis and induces *TPH*2 expression by cortisol released from stress-activated adrenal secretions, which enhances 5-HT and inhibits adrenal cortisol secretion by ACTH [10].

Estrogens are mainly produced in the ovaries, the placenta in females, and the adrenal cortex of males, and are retained in target cells by estrogen receptors (ERs) [83]. Four estrogens, estrone (E1), 17β-estradiol (E2), estriol (E3), and estetrol (E4), have been identified in humans. There are three types of ERs, classical ERα, ERβ, and non-classical G protein-coupled estrogen receptor 1 (GPER1). ERα and ERβ are encoded by *ESR1* on chromosome 5 and *ESR2* on chromosome 14, respectively. ERα and ERβ exhibit a high degree of homology. ERβ is expressed in the brain, cardiovascular system, lung, and immune system, whereas ERα in the uterus, ovary, bone, white adipose tissue, and liver.

Estrogens bind and change ER conformation, allowing it to interact with specific estrogen response elements (EREs) on the promoter of the target gene, and modulate gene transcription. In classical pathways, ERs form homo- or hetero-dimers, and directly interact with ERE [84,85]. ERs interact with transcription factors, such as AP-1, simian virus 40 promoter factor 1 (Sp1), CCAAT/enhancer binding protein β (C/EBPβ), and nuclear factor κB (NF-κB), and indirectly activate transcription [86,87]. E2 activates tyrosine kinase (SrcK)/MAPK and PI3K/AKT, signaling pathways, phosphorylates, and activates certain nuclear transcription factors.

Interaction between 5-HT and estrogen is indicated by the colocalization of both systems in the same brain regions and is involved in the pathophysiology of depression, migraines, irritable bowel syndrome, eating disorders, and pregnancy-related pathologies [88,89]. Estrogen activates the nuclear and membrane ERβ and induces *TPH2* expression through an ERE half-site located within the *TPH2* promoter’s 5′-untranslated regions [90]. Estrogen and progesterone increased *TPH2* mRNA in the dorsal raphe region of female macaques [91], and estrogen increased *TPH2* in the rat raphe nuclei and ameliorated anxiety-like behavior [92]. In female and male ERβ -knockout (KO) mice, 5-HT levels decreased significantly in the hippocampus and nucleus accumbens, and mice showed anxiety-like behavior [93,94]. Estrogens exert antidepressant activity via multiple mechanisms: the regulation of 5-HT synthesis, metabolism and function, and neurotrophic activity to promote neuroplasticity and neurogenesis [95]. Estrogen increases the expression of *TPH2*, *TH*, and *SLC6A4*, downregulates MAO and the 5-HT1A receptor and presents antidepressant-like effects in female patients with depression [79]. E2 increased 5-HT2A and SERT in the thalamus and hypothalamic nucleus of female ovariectomized rats [96]. E2 increased the 5-HT transporter in the superior frontal cortex, anterior cingulate cortex, nucleus accumbens, and 5-HT2A receptor in the frontal cortex and striatum of ovariectomized *Macaca fascicularis* [97,98].

Estrogen influences neural network formation in the SN and ventral tegmental. The *TH* promoter contains *AP-1*/*early growth response gene 1* (*Erg*) motifs, which are required for E2-mediated *TH* induction by ERβ, but not ERα [99,100,101]. Estradiol increased *TH* and *DBH* levels in the locus coeruleus and nucleus solitarius, but not in the SN of ovariectomized female and castrated male rats [101,102,103,104]. E2 increased *TH* promoter activity and expression in the locus coeruleus of female mice but decreased them in males [105]. E2 interacted with ERα at the plasma membrane, activated PKA or MAPKs, phosphorylated CREB, modulated CRE-mediated *TH* transcription in PC 12 cells [106], and increased catecholamine synthesis in cultured bovine adrenal medullary cells [107,108].

**Figure 5:**
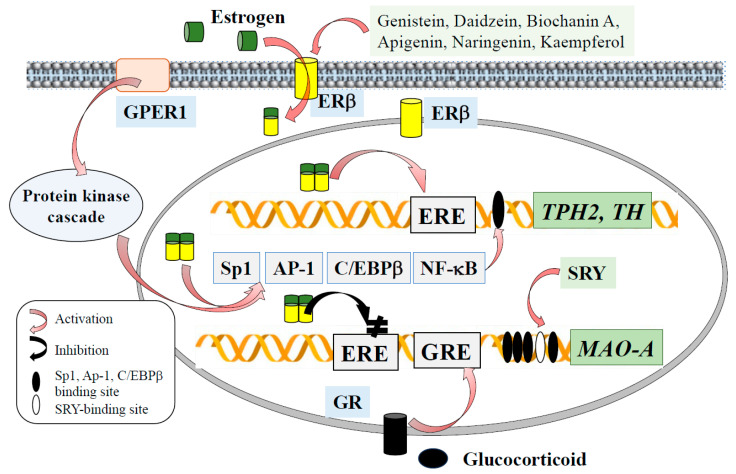
Estrogen binds to ERβ, forming a complex that activates ERE at the *TPH2* or *TH* promoter, or binds to Sp1, AP-1, CCAAT/enhancer binding protein b (C/EBPβ), or NF-κB, then to their binding sites, promotes the expression. Estrogen binds to GPER1, activates protein kinases cascade and transcription factors. Estrogen binds ERE and downregulates *MAO-A* transcription. Sex-determining region also regulates *MAO-A* transcription. Stress increased glucocorticoid and activated *MAO-A* expression. Phytoestrogens with high affinity to ERβ are shown.

Estrogen binds to ERβ, interacts with ERE in the *MAO-A* promoter and downregulates *MAO-A* expression. MAO-A is associated with sexual dimorphism in several neuropsychiatric disorders. The high prevalence of depression and dementia in peri- and post-menopausal women suggests the modulation of monoamine metabolism by estradiol [109]. MAO-A, assessed by [^11^C]-harmine (a β-carboline MAO-A inhibitor) position computed tomography (PET) imaging, increased by 43% in the brain of postpartum women, whose estrogen levels dropped by 100–1000-fold during the first 3 to 4 days [110]. In perimenopausal women, MAO-A increased by 34% and 16% compared with reproductive and menopausal women, respectively [111]. Estrogen significantly decreased MAO-A activity in the hypothalamus (−28%) and amygdala (−21%) of adult female ovariectomized rats, whereas MAO-B activity in the brain did not change [112]. ERβ regulates *MAO-A* expression in the dorsal raphe and paraventricular nucleus, ERα regulates *MAO-B* in the preoptic area, and both ERα and ERβ regulate *MAO-A* and *MAO-B* expression in the ventromedial nucleus [113]. The *MAO-A* promoter consists of the Sp1 binding site and sex-determining region Y (SRY) binding site [114]. Sp1 and Sp4 directly interact with Sp1 sites and activate the *MAO-A* and *MAO-B* promoters, whereas Sp3 inhibits them by competition for binding to the CACCC element [115] (Figure 5). Androgen and glucocorticoid upregulate *MAO-A* expression, through binding to the androgen receptor (AR) or glucocorticoid receptor (GR), and direct interaction with the functional androgen/glucocorticoid response element (ARE/GRE) [116]. High-dose testosterone treatment decreases MAO-A in human brains [117]. E2, estrogen metabolite, and 4-hydroxeuilenin (4-OHEN) downregulate *COMT* expression and are substrate sand inhibitors of COMT in MCF-2 cells [118,119,120].

Exposure to stressors causes an increased release of monoamine neurotransmitters in the limbic structures and activation of the HPA-axis, leading to an increase in circulating glucocorticoids. Glucocorticoids bind and activate GRs, and subsequently interact with GRE on the targeted gene. *TPH2* exhibits flexible gene expression in response to stressful events or stressors [10]. The 5′-untranslated region of *TPH2* and *TH* contains a binding motif, the repressor element-1 (RE1) for the repressor element-1 silencing transcription factor (REST). This motif functions as an “on-off” switch for *THP2* and *TH* transcription [121]. Glucocorticoids modify *TPH2* expression and 5-HT synthesis in a species-specific way. In the raphe nuclei of ovariectomized female and intact male mice, dexamethasone decreased TPH expression [122], whereas in rats, glucocorticoids induced *TPH* expression and TH synthesis [123]. A daily fluctuation of glucocorticoids regulates circadian rhythmic *TPH* expression in the raphe nucleus. Stress induces *TH* and *DBH* expression in the sympathetic ganglia and adrenal medulla, depending on stress type, animal species, and target tissues in animal models of stress [124]. A glucocorticoid response element was reported at AP-1-like sequences on the rat *TH* gene [125]. Chronic stress-induced glucocorticoids increased *Kruppel-like factor 11* [*KLF11*, also called *transforming growth factor β-inducible early gene* (*TIEG2*)], translocated KLF11 from the cytoplasm to nucleus, and activated Sp/KLF-binding sites at the *MAO-A* promoter and increased the expression of MAO-A in the rat brain [126].

## 5. Phytoestrogens Promote Expression and Activity of Tryptophan Hydroxylase and Tyrosine Hydroxylase

The discovery of ERβ has provoked the search for ER type-specific ER modulators (SERM) for the prevention or treatment of menopausal symptoms, osteoporosis, breast cancer in women, and other estrogen-related disorders. ERβ-selective SERM (coumestrol, diarylpropionitrile) administration in the hippocampus of ovariectomized rats decreased anxiety and depressive behaviors [127]. However, ERβ-targeted SERM has not been available in practice, and applications of plant bioactive compounds have been searched for to prevent neuropsychiatric disorders, cardiovascular disease, cancer, and metabolic syndrome [128]. Phytoestrogens are nonsteroidal estrogenic polyphenols derived from dietary plants. They include isoflavones (daidzein, genistein, biochanin A), flavonoids (chrysin, apigenin, apigenin, naringenin, kaempferol, quercetin), stilbenes (*trans*-resveratrol), coumestrol, and lignans. Phytoestrogens have been proposed as part of hormone replacement therapy in estrogen-related disorders [129]. Soybean-derived isoflavones have some benefits for menopausal syndromes, such as vasomotor syndromes and hot flashes, but clinical trials could not confirm the effects on menopausal syndromes [130,131].

Phytoestrogens bind ERs at the membrane and nucleus, induce estrogen-dependent gene transcription, and have biological activity similar to E2 [18]. Some of them (coumestrol, genistein, apigenin, naringenin, kaempferol) have a higher binding affinity to ERβ than ERα and stimulate transcriptional activity [132,133]. Among flavonoids, genistein (4′,5,7-trihydroxyisoflavone) exerts the most potent estrogenic activity to ERβ, followed by daidzein (4′,7-dihdroxyisoflavone), biochanin A (4′,5-dihdroxy-7-methoxyisoflavone), apigenin (4′,7-dihdroxyflavone), kaempferol, naringenin, quercetin, and chrysin. The position and number of the hydroxyl substituents on the flavone or isoflavone molecule determine the ER binding affinity. 8-Prenylnaringenin, biochanin A, daidzein, genistein, naringenin, resveratrol, and quercetin modulate plasma estrogen levels in pre- and post-menopausal women and male/female volunteers [134]. (In the Supporting Information, Figure S1 presents the chemical structure of estrogenic phytochemicals).

Phytoestrogens modulate the serotonergic and dopaminergic systems, and improve brain function and neuropsychiatric diseases [15]. They affect the activity and expression of TPH, TH, and MAO and regulate the monoamine biosynthesis, metabolism, and function. In addition, phytochemicals control the transporters and receptors of monoamine neurotransmitters. In an aged brain, oxidative stress and inflammation decreased TPH and TH phosphorylation and their activities [135,136]. Silymarin, quercetin, naringenin [137], catechin, and tea extract (polyphenon-60, a catechin extract) [138] enhanced the activity of TPH and TH in the brain and adrenal medullary cells. Chronic resveratrol treatment (20 mg/kg/day for 4 weeks) in old male rats (20 months) increased the activity of TPH2 (70–51%) and TH (150–36%) in the hippocampus and striatum; TPH1 activity (463%) in the pineal gland; enhanced 5-HT, NE, and DA levels; and improved cognitive and motor functions [139]. Korean red ginseng increased *TPH2* and *TPH1* in the hippocampus of a rat model of prolonged stress and ameliorated depression-like behavior [140,141,142]. Anthocyanin extract from blueberries increased the TPH protein in the hippocampus of aged male rats [143].

Puerarin extracted from *Pueraria lobata* increased TH expression in the SN by about 85% in rats [144]. Sesamol and naringenin increased TH expression in the SN of a rotenone-treated PD rat model [145]. Chlorogenic acid, a *trans*-cinnamic acid ester, increased *TH* expression in the SN and striatum of MPTP-treated mice [146]. Resveratrol increased TH protein expression in the striatum of pups exposed to lipopolysaccharide in utero from dams fed with a resveratrol-supplemented diet [147]. Phloretin (dihydronaringenin) found in apples increased *TH* protein expression in the striatum of MPTP-treated mice and protected DA neurons by anti-inflammatory activity [148]. Tangeritin, a citrus polymethoxy flavone, increased TH expression in a Drosophila model of PD [149]. Curcumin increased expression of *TH* mRNA, DA, and NE levels and downregulated MAO in the limbic system and midbrain of ovariectomized rats [150]. The non-planar structure and hydroxy groups on aromatic rings of curcumin seems to promote *TH* transcription. Gardenin A, a polymethoxy flavone isolated from *Cardenia resinfera*, regulated the nuclear factor erythroid 3-related factor (Nrf2), and increased TH protein expression in the striatum of A53T α-synuclein-overexpressing mice [151].

Daidzein and resveratrol activated TH activity in cultured adrenal medullary cells through ERK1/2 activation [19]. Daidzein at low concentrations enhanced catecholamine neurotransmitter synthesis via binding to plasma membrane ERs, but at high concentrations, inhibited the biosynthesis [152]. Nobiletin, a citrus polymethoxy flavone, phosphorylated Ser^19^ and Ser^40^ of TH and increased TH activity in cultured bovine adrenal medullary cells by PKA [153]. (In the Supporting Information, Table S2 presents the chemical structure and function of phytochemicals affecting TPH and TH).

## 6. Phytochemicals Inhibit Expression and Activity of Monoamine Oxidase and Catechol-*O*-Methyltransferase

MAO plays crucial physiological roles and MAO-A is a target for the therapy of depression and anxiety, whereas MAO-B for that of PD and AD. Synthetic, irreversible MAO-A inhibitors (clorgyline) showed critical side effects, and reversible MAO-A inhibitors (moclobemide, brofaromine, toloxatone) are clinically applied for the treatment of anxiety and depression. Irreversible MAO-B inhibitors, selegiline [(−)-deprenyl] and rasagiline and their derivatives, are used for the treatment of neurodegenerative diseases, including PD, AD, and aging. Herbs and herb preparation inhibit the activity of MAO and COMT, and are considered as effective alternative therapies in neuropsychiatric diseases [154]. Herb ingredients, including flavonoids (flavanols, flavanones, flavones, isoflavones flavonols), alkaloids, chalcones, coumarins, and xanthones (mangiferin), have been proposed to inhibit MAO-A and exert antidepressant-like and neuroprotective functions [155,156].

Quercetin and structurally related flavonoids can cross the BBB, and attenuate MAO-A in the brain to exert antidepressant-like effects in rodent models of depression [157]. Chemical structures for specific MAO-A inhibition by flavonoids are indicated. Hydrophobicity and the planar structure of the diphenyl-propane (C_6_-C_3_-C_6_) skeleton are required for effective inhibition against MAO-A. A flavone with a planar structure inhibits MAO-A better than a flavanone with a nonplanar skeleton [158]. Hydroxy residue at the C-4′ position of the B ring of flavonoids increases the affinity to MAO-A [159]. Quercetin, kaempferol, purpurin, and apigenin are potent, selective, reversible, competitive MAO-A inhibitors [160,161], whereas chrysin is a potent MAO-B inhibitor [162]. Alizarin (1,2-dihydroxyanthracene-9,10-dione) inhibited MAO-B, and less potentially, MAO-A. Resveratrol and (−)-*trans*-e-viniferin (a resveratrol dimer), natural coumarin, xanthotoxin, and praeruptorin-A have MAO-A and -B inhibition and antidepressant activity [163]. Xanthines (caffeine), flavonoids (gancaonin A), protocatechuic acid, and alkaloids (piperine) are potent reversible, selective MAO-B inhibitors [164]. (Figure S2 presents the chemical structure and function of phytochemicals affecting MAO in the Supporting Information).

Polyphenols are used as scaffolds for the development of new MAO inhibitors. Open chain flavonoids composed of a core structure of 1,3-diaryl-2-prone-1-one are derivatized as MAO-A and -B inhibitors. Derivatives of natural and synthetic chalcone (1,3-diphenyl-2-propaen-1-one) inhibit MAO, whereas those of heterocyclic (furan, thiophene, piperidine, quinoline) are potent, reversible MAO-B inhibitors [165]. 3-Phenylcoumarin derivatives, such as aminopyran, are synthesized to develop new antidepressants [166]. The alkyl-sulfonyl group substitution at the C-7 position of coumarin provided MAO-A inhibiting activity in esuprone and 7-oxycoumarin [167,168,169]. A benzyloxy substitution increased selective MAO-B inhibition in 7-benzyloxy-3,4-dimethlcoumarin and 3-arylcoumarin derivatives [168,170].

Several natural and synthetic COMT inhibitors have been developed to increase the available monoamine neurotransmitters for therapy for PD, depression, and schizophrenia [170]. The derivatives of pyrogallol and catechol, such as gallic acid, caffeic acid, 2-hydroxy estradiols, or flavonoids (quercetin, rutin), are so-called “first-generation” COMT inhibitors. Derivatives of nitrocatechol, chalcone, and pyrazoline have been developed as the “second generation” of COMT inhibitors [171]. Entacapone and opicapone have been synthesized for adjunctive therapy of PD. COMT inhibitors with low toxicity and high bioavailability have been searched among catechol and DA derivatives. Catechol polyphenols, (+)-catechin, alizarin, morin, chlorogenic acid, and fisetin inhibited COMT in rat liver [172]. Chlorogenic acid [173] and catechins with a galloyl-type D-ring, EGCG, and (−)-epicatechin-3-gallate were the most potent human liver COMT inhibitors [174]. (Figure S3 presents the structures of cited compounds that inhibit COMT in the Supporting Information).

## 7. Epigenetic Phytochemicals Affect Monoamine Neurotransmission and Prevent and Treat Monoamine-Related Disorders

Neurodevelopment is under continuous endogenic and exogenic stress from gestation to adulthood. Life experiences influence behavior, stress response, and disease susceptibility through the epigenetic modification of expression of genes, and subsequently, enzymes and molecules [175]. Nutrients and bioactive food components show epigenetic modification, and an “epigenetic diet” has been proposed to delay the onset of aging and age-related neurodegeneration [176]. The epigenome begins encoding in the uterus and epigenetic mechanisms are associated with neurogenesis, cell migration, and synaptogenesis in early brain development [177]. Periconceptional nutrients can affect the epigenomic state of offspring during the first 1000 days, from conception to the end of age 2, and significantly affect neurodevelopment. The perinatal nutritional status causes permanent changes in insulin-like growth factor 2 (IGF2) methylation and the newborn’s fetal growth [178]. The maternal intake of flavonoid (kaempferol-3-*O*-glucoside, narirutin) reverted depression-like behavior in female rat offspring [179]. Dietary phytochemicals may modify the epigenome, intervene in the development of monoamine-related disorders, and help prevent their progression.

Polymorphisms of *TPH2*, *SERT*, and *MAO-A*, and exogenous factors, such as stress, physical abuse, nutrition, tryptophan depletion, and poor maternal care, influence 5-HT systems. 5-HT-dependent signaling regulates the early phase of brain development [180]. In the human brain, 5-HT neurons are identified by 5 weeks of gestation ahead of other monoamine systems, and proliferate until gestational week 10 [181] and regulate the development of other neurotransmitter systems [1]. 5-HT levels increase during the first 2 years of age and decline to adult levels by age five. Decreased or increased 5-HT levels affects the structure of brain circuits and is implicated in psychiatric disorders [182,183]. Prenatal treatment with 5,7-dihydroytryptamine decreased 5-HT signaling, induced maladaptive behaviors, including depressive-like and anxiety-like behavior, and defective social interactions in adult rats [20]. Licking/grooming in rats increased 5-HT, activated the 5-HT7 receptor, promoted NGF-inducible factor A (NGFI-A), demethylated the GR promoter, and modulated the HPA response to stress [184]. The depletion of 5-HT decreased neurogenesis, induced abnormal dendritic density in the hippocampus, and impaired spatial learning [185]. Excess 5-HT during the early stage disrupts the normal wiring of the somatosensory cortex. In *MAO-A* or *SERT* KO mice, the thalamocortical axons failed to segregate and did not form a normal barrel-like structure [186]. 5-HT excess caused by a *MAO-A* deficit is involved in antisocial and aggressive behavior, as shown in Brunner syndrome and *MAO-A* KO mice [20]. In *MAO-A* KO mice, forebrain-expression of *MAO-A* reduced 5-HT, NE, and DA levels, restored the brain structure, and rescued aggressive behavior [187].

**Figure 6:**
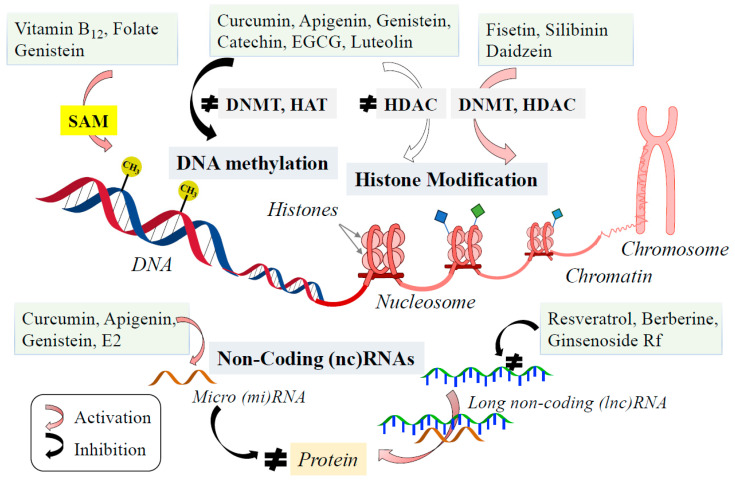
Mechanism of diet components and phytochemicals in the regulation of epigenetic modification. Folate, vitamin B_12_, and genistein increase SAM availability. Curcumin, apigenin, genistein, catechin, EGCG, and luteolin inhibit DNMT, HAT and HDAC. Fisetin, silibinin, and daidzein activate HDAC, leading to regulate gene expression. Some phytochemicals upregulate miRNA or downregulate lncRNA.

Epigenetic changes are mediated by DNA methylation, histone modification, non-coding RNA (ncRNA), and a changed structure of chromatin (Figure 6). DNA methylation occurs on cytosines at CpG sites by DNA methyltransferases (DNMTs) using SAM as a methyl donor, and represses gene expression. In mammalian cells, the *N*-terminal histone tails are post-translationally modified by acetylation, methylation, phosphorylation, and ubiquitination. Histone acetylation is controlled by histone acetyltransferases (HATs) and histone deacetylases (HDACs). HATs add chromatin acetyl groups, make chromatin conformation more relaxed, and promote gene transcription, whereas HDACs act in reverse ways. ncRNAs are classified into microRNAs (miRNAs) and long noncoding RNAs (lncRNAs) by their length. miRNAs are small, single-stranded RNAs with around 22 nucleotides in length, bind to the target mRNA at the 3′-untranslated regions (3′UTRs), degrade, destabilize mRNA, inhibit translation, and silence gene expression. lncRNAs are RNAs with more than 200 nucleotides in length, modulate gene expression at the transcriptional and post-transcriptional levels, and modulate chromatin architecture. lncRNAs are strongly expressed in the brain, and more than 3600 brain-specific lncRNAs have been identified, and they play a role in neural development, proliferation, and differentiation as epigenetic regulators [188]. lncRNAs are involved in the onset and development of schizophrenia, MDD, bipolar depression, and suicide [189]. lncRNAs affect depression by interacting with the epigenetic modification of DNA and chromatin, gene SNPs, and compete with endogenous RNA networks related to NTF expression and synaptic function [190].

Epidemiological and preclinical studies have presented that early-life child maltreatment, parent neglect, undernutrition, or sexual abuse increase the risk for depression and other stress-related disorders by two- to four-fold [191,192]. An unfavorable maternal environment induces long-term epigenomic changes in gene expression related to 5-HT that persist into adulthood [193]. In adult males with high childhood-limited aggression, early-life stress promoted methylation levels of the *SLC6A4* promoter in T cells and monocyte, and associated with lower 5-HT synthesis in the lateral orbitofrontal cortex measured in vivo using PET imaging [194]. Increased DNA methylation in the promoter region of *5-HTR1A* gene was observed in schizophrenia and bipolar disorder [195]. A study on the *5-HTT* methylation status in 10-year-old monozygotic twins presented that 5-HTT levels were significantly higher in the twin who experienced bullying and stress than the one who did not undergo such experiences [196]. MAO-A expression and activity are negatively correlated with the methylation status of the *MAO-A* promotor at CpG islands (CGIs) including 14 CpG sites [197].

Sulforaphane, flavonoids, resveratrol, and curcumin can reverse abnormal gene expression by epigenomic mechanisms, and exert therapeutic potential for neuropsychiatric disorders, aging, cancer, and chronic inflammation [198,199,200]. The impact of diets on epigenetic regulation is mainly the supplementation of methyl groups from methionine, vitamin B_12_, folate, betaine (trimethylglycine), and polyphenols (genistein) [201]. Curcumin, genistein, apigenin, luteolin, catechins, and EGCG activate NF-κB expression, inhibit DNMT and HDAC, and remodulate chromatin conformation and reverse abnormal gene expression. A hydroxyl group at the C-7 position in ring B of fisetin (3,7,3′,4′-tetrahydroxyflavone), silibinin, and daidzein are required for the activation of DNMT and SIRT1, whereas a hydroxyl group at the same position in luteolin and EGCG inhibits SIRT1. Genistein, myricetin, and quercetin with hydroxyl groups at positions C-5 and C-7, inhibit or activate HDACs and DNMTs depending on experimental conditions [202]. Quercetin, icariin, silibinin, daidzein, formonetin, and biochain A increased *SIRT1* and *PGC-1α* expression, increased AMPK phosphorylation, induced histone deacetylation, and exerted neuroprotection in cellular models of neurodegenerative diseases. Polyphenolic HDAC activators, such as quercetin, halted or delayed the propagation of aging and significantly prolonged the life span of mice through the inhibition of SIRT1 by quercetin-3-*O*-gluconide, a quercetin metabolite [203]. Curcumin increased miR-128 and miR-9 and downregulated phosphorylated tau in rat cortical neurons [204]. Apigen increased miR-15a expression, decreased Rho-associated protein kinase-1 (ROCK-1), and exerted neuroprotection in the rat hippocampus [205]. Genistein and E2 enhanced miR-132, BDNF, and IGF-1 in the hippocampus of ovariectomized rats and improved spatial memory [206]. Resveratrol downregulated lncRNAs (SNHG1, MALAT1), and upregulated the miRNAs (miR-361 -3p, miR-129) and TH cells in cell models of AD and PD [207,208]. Berberine downregulated BACE1-AS and LINC00943, increased miR-129 and miR-142n, and showed neuroprotection and anti-inflammation and anti-apoptosis activity [209]. Ginsenoside Rf modulated the expression of lncRNAs (Mett127, Cyp23, MSN, Ptx3, et al.) and inhibited tau aggregation and toxicity [210].

Epigenomic phytochemicals have been clinically tried as attractive therapeutic agents for cancer, aging, cognitive decline, obesity, and chronic inflammation [211,212]. In healthy premenopausal women, isoflavones (40 or 140 mg, daily, through one menstrual cycle) increased the methylation of breast cancer-related genes *RARb2* and *CCND2* in correlation with serum genistein levels [213]. The administration of genistein (54 mg daily for 2 years) in postmenopausal women decreased the depression score, which was assessed with the Zung Self-rating Depression Scale [214]. Polyphenols, saponins and terpenoids, and alkaloids have been proposed as antidepressant candidates without severe side-effects by the upregulation of 5-HT, NE and DA, BDNF induction, MAO inhibition, and the suppression of HPA axis overactivity [215]. The efficacy of phytochemicals as antidepressants has been presented by preclinical investigations and some clinical investigations [216]. Curcumin administration reduced depressive symptoms in patients with MDD [217].

## 8. Clinical Applications of Phytochemical Treatment in Neuropsychiatric Disorders

The malfunction of the 5-HT system, including TPH, 5-HT1, 5-HT7, and SERT, is involved in mood disorders (depression, anxiety), cognition decline, and neurodevelopmental disorders. There is a critical window in a distinct stage of brain development, during which 5-HT levels permanently regulate neuronal architecture [218]. Can phytochemicals prevent or ameliorate the 5-HT-related impairment of brain development during early stage? Preclinical evidence suggests that dietary and bioactive phytochemicals can influence monoamine biosynthesis, metabolism, and function, with certain compounds, particularly phytoestrogens, also exhibiting estrogen- and BDNF-like activities. These properties may contribute to their neuroprotective effects via epigenetic modifications, highlighting their potential role in modulating neurodevelopment and neurotransmitter function. Many neuroprotective phytochemicals, including alkaloids, polyphenols, and terpenoids, have demonstrated promising in vivo and in vitro effects but remain largely unexplored in humans. Recent meta-analyses have highlighted several important compounds, including huperzine A, caffeine, curcumin, resveratrol, quercetin, ginkgolide, ferulic acid, rosmarinic acid, as potential therapeutic agents across conditions such as depression, anxiety, schizophrenia, AD, and dementia [25,219,220,221] These compounds have progressed to clinical trials [221,222].

While these findings support their potential as therapeutic agents for monoamine-related diseases, direct clinical evaluations remain limited due to multiple challenges in clinical application. Neuropsychiatric disorders involve complex, multifactorial systems, making it difficult to establish clear therapeutic targets. Additionally, the diverse and heterogeneous nature of these disorders contributes to inconsistent clinical outcomes, sometimes yielding contradictory results that require more reliable and reproducible evidence [223,224]. Accessibility issues, ambiguous effectiveness, and potential side effects further complicate their translation into clinical use [225]. Moreover, phytochemicals are frequently administered as adjunctive or complementary therapies, often in the form of plant extracts or multi-component formulations, making it challenging to isolate and assess the effects of individual compounds [226,227]. Alongside these factors, poor bioavailability, limited blood-brain barrier permeability, and variability in assessment methods further hinder their clinical validation [228].

One problem is how to effectively evaluate the effects of phytochemicals on monoamine systems in vivo. Monoamine neurotransmitter concentrations can be measured in cerebrospinal fluid (CSF) to monitor their synthesis, metabolism, and the activity of the related enzymes. The levels and activity of monoamine transmitters can be indirectly visualized using PET or single-photon emission computed tomography (SPECT) [229]. For example, the 5-HT levels in children with autism could be determined by measuring SERT expression using SPECT with [^13^1I]-*N*-(2-fluoroethyl)-2b-carbomethoxy-3b-(4-iodophenyl)-nortropane [230]. In humans, MAO level and activity are measured using PET imaging with [^11^C]- or [^18^F]-radiotracer aliphatic amines and MAO inhibitors, commonly harmine [231,232]. These advanced imaging techniques offer a powerful, non-invasive approach to monitoring monoamine neurotransmission; however, optimizing their cost, accessibility, and technical feasibility is essential for broader clinical application.

Another key limitation in the clinical application of phytochemicals for neuropsychiatric diseases is their poor bioavailability and stability due to extensive metabolism. In the digestive system, phytochemicals undergo hydrolysis, microbial degradation, hepatic metabolism, and rapid excretion, significantly reducing their systemic availability. In humans, only a small part of ingested polyphenols (0.3–43%) are found in urine, indicating their poor bioavailability [233]. To overcome this, various nanotechnology-based delivery systems, such as solid lipid nanoparticles (SLNs), chitosan nanoparticles, and polymeric nanocarriers, have been developed to enhance absorption, improve stability, and promote CNS targeting for compounds like genistein and curcumin [234,235]. Additionally, alternative administration routes, including the intranasal delivery of genistein-loaded chitosan nanoparticles and transdermal administration of resveratrol-loaded SLNs (RSV-SLNs) via microneedle patches, have been explored to bypass first-pass metabolism and enhance systemic retention [235,236,237]. Prodrug approaches, such as the conversion of curcumin to a curcumin diethyl γ-aminobutyrate (CUR-2GE) prodrug has been shown to enhance its bioavailability and anti-neuroinflammatory properties [238]. Curcumin–piperine-loaded self-nanoemulsifying drug delivery systems (SNEDDS) have presented a safe and effective oral delivery method for improving curcumin bioavailability and brain targeting in AD models [239].

Once phytochemicals achieve sufficient systemic circulation, the next challenge is crossing the BBB to exert neuropsychiatric effects. The restricted permeability of the BBB limits the therapeutic efficacy of many phytochemicals, as only compounds with optimal lipophilicity, molecular size, and transport mechanisms can effectively reach neuronal targets [240] The brain entry of polyphenols depends on their stereochemistry, lipophilicity, and interactions with efflux transporters, such as P-glycoprotein (PGP) [241]. Many flavonoids, flavones, flavonols, isoflavones, phenolic acids, and stilbenes are ligands for brain efflux transporters and penetrate the brain to varying degrees [242]. Lipophilic modifications, such as *O*-methylation and esterification, enhance BBB penetration [243]. Lipid-based and mucoadhesive systems, including glucose-modified liposomes targeting the glucose transporter 1 (GLUT1), have demonstrated increased BBB permeability and neuroprotection of quercetin in oxidative stress models [244]. Curcumin-loaded exosomes have been investigated as a strategy to facilitate BBB crossing, enhancing its neuroprotective effects [245]. Additionally, borneol, a natural monoterpene, has been investigated for its ability to transiently increase BBB permeability, thereby improving the brain bioavailability of co-administered neuroprotective agents. It modulates efflux transporters, alters tight junction proteins, and enhances vasodilatory neurotransmitters, facilitating a greater CNS uptake of therapeutic compounds [246].

While phytochemicals hold therapeutic potential in neuropsychiatric diseases, their clinical application remains limited by many challenges. Advances in formulation strategies, biotechnologies, and permeability enhancers like borneol provide promising solutions to these limitations, but research has largely focused on a few well-studied compounds like curcumin, resveratrol, and quercetin. Expanding research efforts and clinical validation to a wider range of phytochemicals is crucial to fully realize their therapeutic potential in monoamine-related psychiatric diseases.

## 9. Conclusions

This review explored the role of phytochemicals in modulating 5-HT, DA, NE biosynthesis, metabolism, and function, highlighting their potential in treating neuropsychiatric disorders. While challenges such as limited bioavailability and BBB permeability remain, advances in nanotechnology and targeted delivery systems offer promising solutions. Notably, phytochemicals and their derivatives act as potent regulators of MAO-A, which, due to its flexibility to environmental changes, plays a more dynamic role in maintaining monoamine neurotransmission than THP and TH [77]. Beyond therapy, the intervention of phytochemicals in the 5-HT-dependent regulation of early brain development may be a novel strategy for prevention and treatment psychiatric disorders, including aggression, depressive disorders, and anxiety.

## Supplementary Materials

The supporting information can be downloaded at: https://www.mdpi.com/article/10.3390/ijms26072916/s1.

## Abbreviations

AP-1	activating protein-1
BH_4_	(*6R*)-*L*-tetrahydrobiopterin
CREB	CRE binding protein
ΔΨm	mitochondrial membrane potential
E2	17β-estradiol
ER	estrogen receptor
ERE	estrogen response element
GTPCH	guanidine triphosphate cyclohydrolase
mPTP	mitochondrial permeability transition pore
NTF	neurotrophic factor
Nrf2	nuclear factor erythroid 3-related factor
PGC-1α	peroxisome proliferator-activated receptor γ coactivator α
SERM	ER type-specific ER modulators
SERT	5-HT transporter
Sp1	simian virus 40 promoter factor 1
SRY	sex-determining region Y
