# Supplementary material for: Phytochemicals Modulate Biosynthesis and Function of Serotonin, Dopamine, and Norepinephrine for Treatment of Monoamine Neurotransmission-Related Psychiatric Diseases
Source: Int J Mol Sci. 2025 Mar 23;26(7):2916. doi: 10.3390/ijms26072916 (PMC11988947; doi:10.3390/ijms26072916)
Supplement: Supplementary file 1 [file ijms-26-02916-s001.zip › ijms-3448364-supplementary.pdf]

Table S1. The chemical structures and functions of phytochemicals affecting mPTP formation.

Chemical Compound	Type	Subtype	Effect on mPTP Formation	Therapeutic Effects/ Potential Applications	References
Ferulic Acid	Phenolic Acid	Non-Flavonoid	Prevents ANT-CypD interaction at the IMM, inhibiting mPTP formation	Antioxidant, anti-inflammatory and neurotrophic effects/ AD, depression, schizophrenia	Wu, Y.; et al. 2017. https://doi.org/10.1007/s00702-016-1624-4.
Astaxanthin	Carotenoid	Xanthophyll	Decreases CypD and ANT expression, prevents mPTP formation	Protective effect of dopaminergic neurons/PD	Krestinina, O.; et al. 2020. https://doi.org/10.3390/antiox9030262.
Resveratrol	Stilbene	Non-Flavonoid	Decreases CypD and ANT expression, downregulates VDAC expression, promotes VDAC dephosphorylation and deacetylation, prevents the mPTP formation	Neuroprotective effects/ Neurodegeneration, age-related cognitive decline	Xi, J.; et al. 2009. https://doi.org/10.1016/j.ejphar.2008.12.024. Liao, Z.; et al. 2015. https://doi.org/10.1002/mnfr.201400730. Tian, M.; et al. 2019. https://doi.org/10.1016/j.ejphar.2018.11.016.
Curcumin	Polyphenol	Diarylheptanoid	Binds to and stabilizes VDAC in the closed state, influencing mitochondrial function, apoptosis, and mPTP regulation	Modulates monoamine neurotransmitters and neuroinflammatory pathway/Depression	Tewari, D.; et al. 2015. https://doi.org/10.1016/j.bbamem.2014.10.014.
Quercetin	Flavonoid	Flavonol	Exhibits concentration-dependent dual effects on mPTP formation, acting as an inhibitor at high doses and an inducer at low doses	Strong antioxidant and anti-inflammatory properties/ Oxidative stress induced disorders	De Marchi, U.; et al. 2009. https://doi.org/10.1016/j.bbabio.2009.06.002.

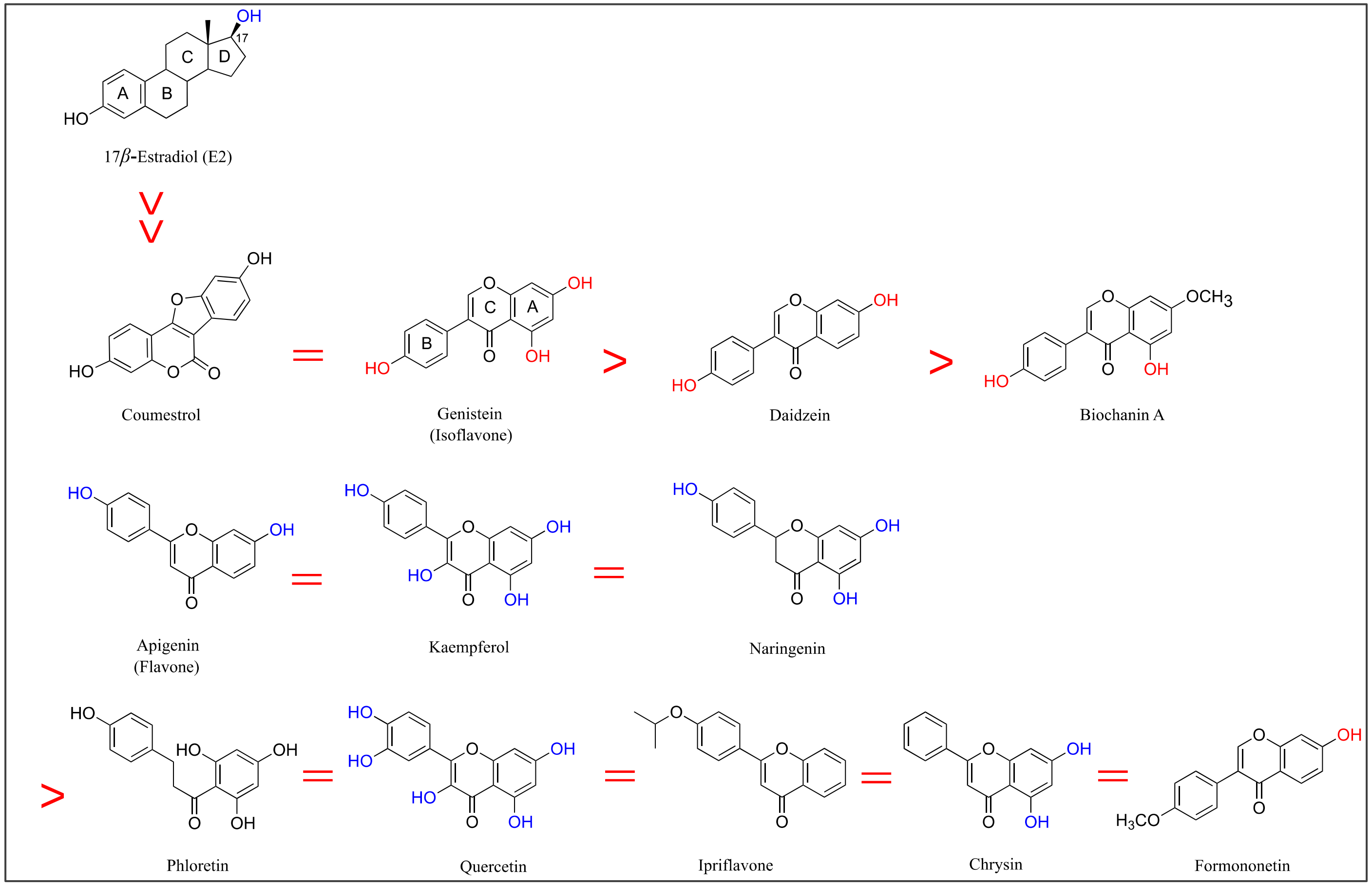

Figure S1. Estrogenic potency of phytochemicals for ERβ (based on Ref. [Kuiper, G.G.J.M.; et al. 1998. https://doi.org/10.1210/endo.139.10.6216.]).

Table S2. The chemical structures and functions of phytochemicals affecting TPH and TH.

Phytochemicals	Structures	TPH	TH	References
trans-Resveratrol	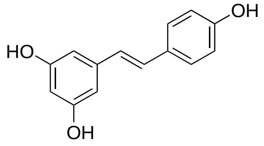	Activity↑	Activity↑<br>Expression↑	Sarubbo, E.; et al. 2015.<br>https://doi.org/10.1007/s11357-015-9777-x.<br>Rose, K.; et al. 2014,<br>https://doi.org/10.1016/j.brainres.2014.05.028.
Silibinin	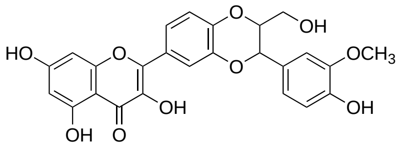	Expression↑		Lee, B.; et al. 2020.<br>https://doi.org/10.1186/s12906-020-2868-y.
Hesperidin	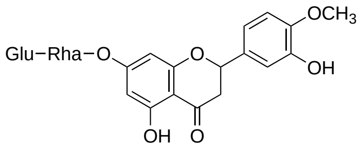	Expression↑		Lee, B.; et al. 2021.<br>https://doi.org/10.1007/s11655-020-2724-4.
Silymarin	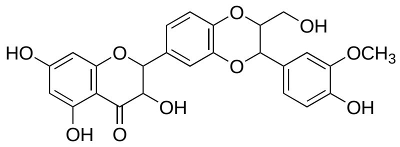	Activity↑	Activity↑	Sarubbo, E.; et al. 2018.<br>https://doi.org/10.1007/s11481-017-9759-0.
Quercetin	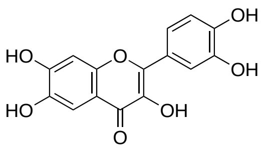	Activity↑	Activity↑	Sarubbo, E.; et al. 2018.<br>https://doi.org/10.1007/s11481-017-9759-0.
Naringenin	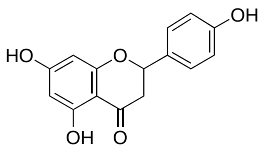	Activity↑	Activity↑<br>Expression↑	Sarubbo, E.; et al. 2018.<br>https://doi.org/10.1007/s11481-017-9759-0.<br>Sonia Angeline, M.; et al. 2013.<br>https://doi.org/10.1016/j.neuroscience.2013.09.029.
Catechin	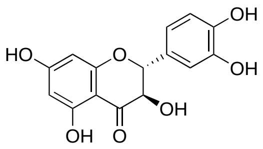	Activity↑	Activity↑	Ramis, M.R.; et al. 2020.<br>https://doi.org/10.3390/nu12020326.

Anthocyanin	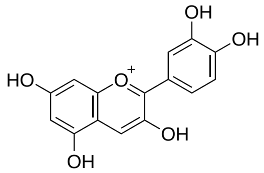	Expression↑		Vauzour, D.; et al. 2021. https://doi.org/10.3390/antiox10081235.
Sesamol	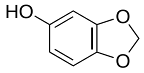		Expression↑	Sonia Angeline, M.; et al. 2013. https://doi.org/10.1016/j.neuroscience.2013.09.029.
Puerarin	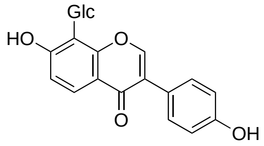		Expression↑	Zhang, X.; et al. 2014. https://doi.org/10.1016/j.neuroscience.2014.08.052.
Chlorogenic acid	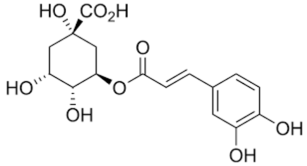		Expression↑	Singh, S.S.; et al. 2018. https://doi.org/10.3389/fphar.2018.00757.
Phloretin	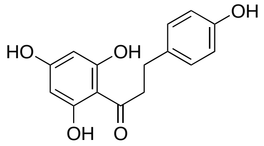		Expression↑	Zhang, G.; et al. 2019. https://doi.org/10.1016/j.lfs.2019.116600.
Tangeritin	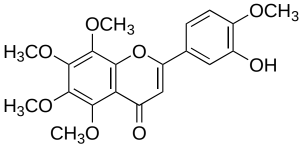		Expression↑	Fatima, A.; et al. 2019. https://doi.org/10.1016/j.neulet.2019.04.047.
Curcumin	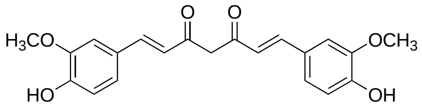		Expression↑	Saied, N.M.; et al. 2021. https://doi.org/10.1111/1440-1681.13427.
Gardenin A	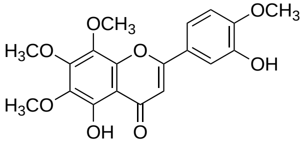		Expression↑	Hack, W.; et al. 2024. https://doi.org/10.1016/j.biopha.2024.116370.

Daidzein	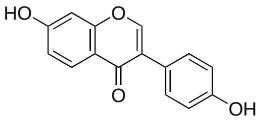		Activity↑	Liu, M.; et al. 2007. https://doi.org/10.1210/en.2007-0073.
Nobiletin	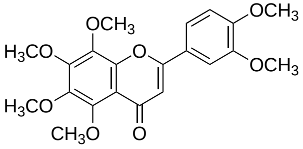		Activity↑	Zhang, H.; et al. 2014. https://doi.org/10.1007/s00210-013-0916-6.

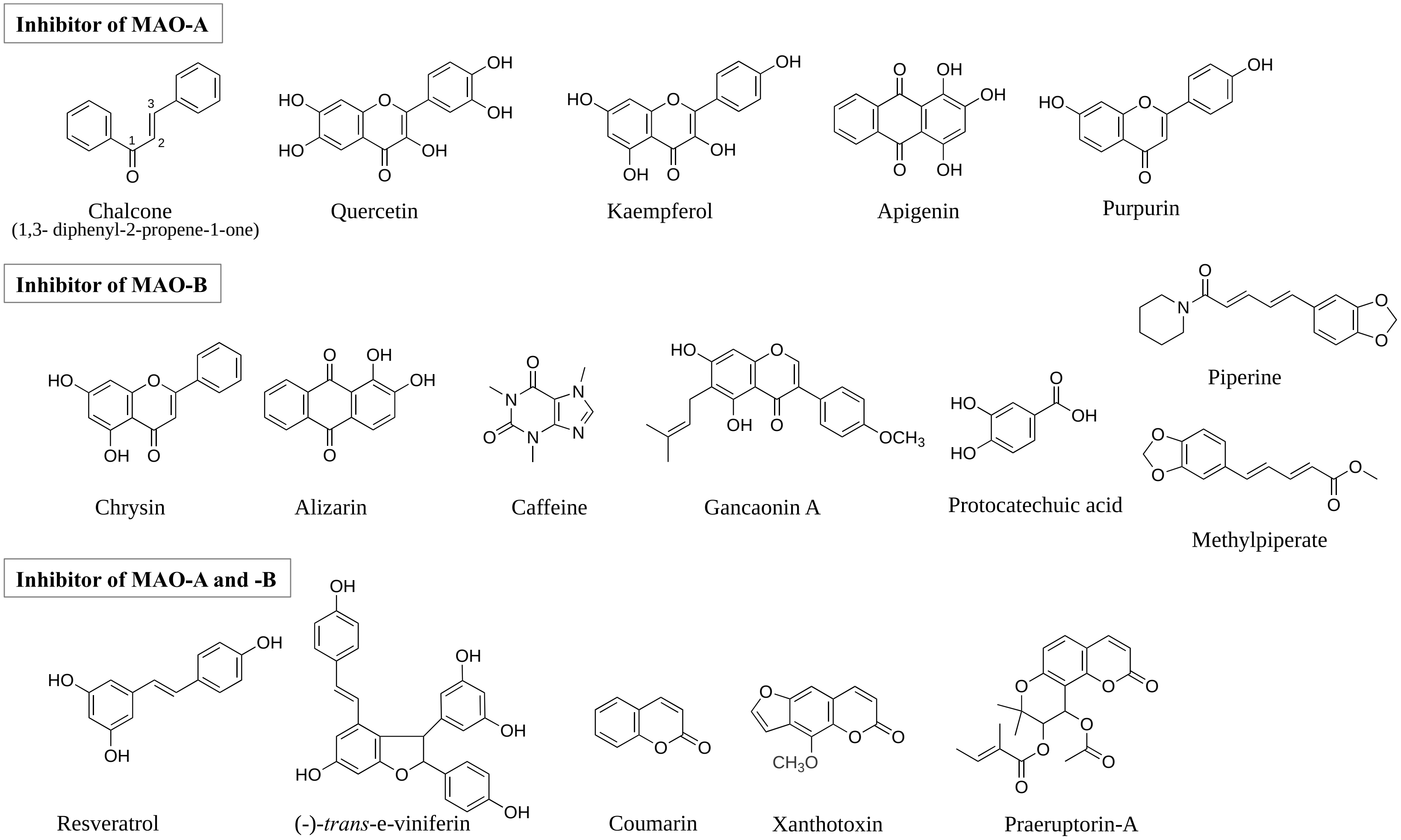

Figure S2. The chemical structures and activities of phytochemicals affecting MAO.

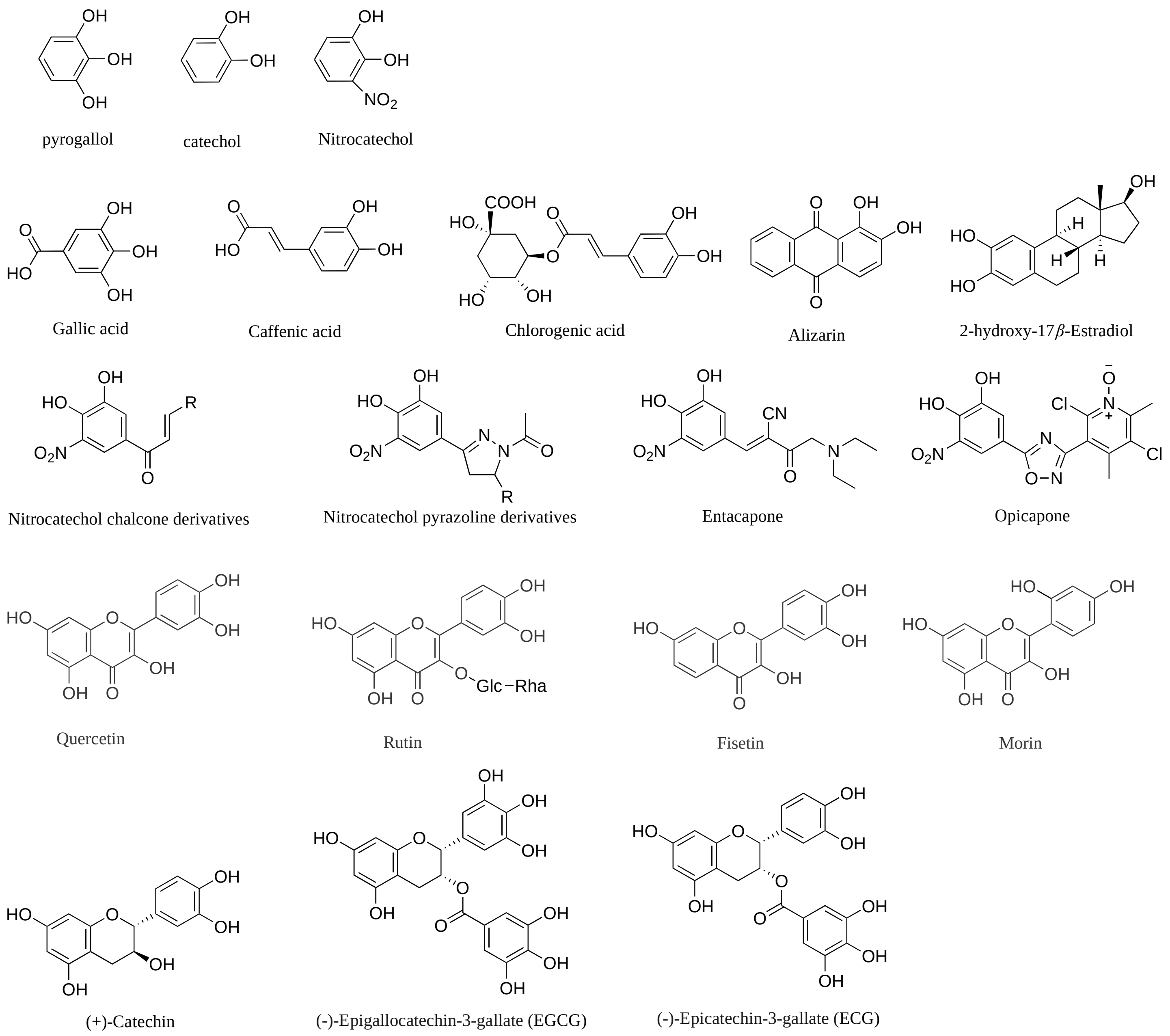

Figure S3. The structures of cited compounds that inhibit COMT.
